# Aneurism of Aorta with Cardiac Hypertrophy

**Published:** 1861-06

**Authors:** 


II. There was presented a case, Pityriasis versicolor, of which the patient had been complaining for three weeks, covering the chest, upper extremities, armpits, and nearly the whole abdomen. It was characterized by the usual yellow color, excessive itching, furfuraceous desquamation, and in the groins by an altered and inflamed condition of the skin.

A lotion of Sulphurate of Potash, and the internal use of powders of Rhubarb, Ipecac and Soda, were prescribed.

III. P. G., aged 33; Scotland; married; of temperate habits; applied at the Hospital for relief, May 18th.

No hereditary tendency to tubercular disease could be traced in his history. *He describes three attacks of rheumatism he has had in the last four years.*

The first commencement of his present difficulty he attribtes to a "a sprain in the chest" he experienced on board a vessel whilst reaching to grasp a rope, three years ago. Shortly afterwards he began to experience severe pain in the right infraclavicular region, which continued steadily for eighteen months, when, in addition to this, he began to suffer from pain in the same region of the left side,—about this time, he became troubled with shortness of breath, whenever he made any unusual exertions, but had no cough. These symptoms continued without much change until twelve months ago, when they were aggravated by a cough, most severe when lying down, and occasionally accompanied with a frothy white expectoration. Last September, the cough became much worse, accompanied with expectoration of blood, at first in clots mixed with a little mucus, and afterwards pure *florid* blood; and this hæmoptysis lasted for eight days. About the 1st of November, the cough began to gradually cease, and again, about the 1st of January, his symptoms became so severe as to confine him to his bed for eight weeks afterwards; the pain being very great across the upper portion of the chest and in the arms, accompanied with fever, dry cough, loss of appetite, and emaciation.

He subsequently improved a little, gained some strength, but the pain and cough, with difficulty of breathing, became much exaggerated in March, and still continues.

He says he is unable to lie on the left side, but does not cough so much at night as he did previously, and the expectoration is mostly white and opaque. He complains of palpitation of the heart, and says he has been subject to it for four or five years, but it has become more constant and troublesome of late; countenance anxious, as one suffering pain; lips bluish; respiration labored and difficult; pulse 130, feeble; appetite poor.

*Physical Examination, First—Inspection.*—The chest is not symmetrical in its proportions, but presenting from the summit of the sternum nearly to the ensiform cartilage, and most markedly on the left, a preternatural fullness, which is seen to pulsate. The apex of the heart beats immediately below the left nipple in the sixth intercostal space. The chest readily expands upon forcible inspiration in a normal manner.

The right infraclavicular region is somewhat more sunken than the left.

*Second—Palpation.*—Upon placing the hand over the prominence before mentioned, a decided hammer-like impulse is imparted to it synchronous with the beating of the heart, and accompanied with a rasping sensation, (*fremissement.*) This can be partially felt just over the sternum, by flexing the head of the patient and forcing the fingers behind it. Vocal vibrations are felt throughout the chest.

*Third—Percussion.*—In the sternal region throughout its entire length, and laterally, an inch from either border, comprising the prominence of the tumor, is dulness amounting to flatness. There is also dulness on percussion under the right clavicle.

The dullness of the left chest anteriorly, commences at the third rib and extends downwards two inches below the left nipple and outwards to a line two inches beyond it. Posteriorly: The right chest is normal on percussion, but the left is dull in the supra and infra-scapular regions—otherwise percussion is normal.

*Fourth—Auscultation.*—The respiration anteriorly, throughout the right chest is puerile, and at its summit approaches in character to that of bronchial, but no moist sounds, broncophony or pectoriloquy, but the attention is attracted by a peculiar churning noise, (*brûit de soufflét.*)

Left chest, anteriorly, respiration weak, (distant); and occasionally accompanied with subcrepitant râles. Under the upper third of the sternum, in addition to the impulse, is heard a double-bellows sound, not unlike an aneurismal brûit.

The apex of the heart gives to the ear the hammer-like impulse before mentioned, and also the two sounds of the heart in such rapid succession that no appreciable interval can be detected, with a slight bellows murmur, commencing with the first sound, but *most distinct* over the *base.* The respiration of the right side, posteriorly, presents no marked peculiarities except being puerile; upon inspiration, the left chest gives some moist sounds.

This double-blowing sound before noticed, is not to be confounded with the sounds of the respiration through the trachea, modified by the impulses of the tumor against it.

*Diagnosis.*—Hypertrophy of Heart; insufficiency of the Aortic valves; and aneurismal dilatation of the arch of the Aorta.

In commenting upon this case, the Professor dwelt upon the affections of the heart and their frequency, as the sequelæ of rheumatism, and especially the insidiousness with which they sometimes approach, as illustrated by the history of this patient—especially when the affection begins in a valvular disease as was probably the fact in this case, though the physical signs are always present to the practiced ear. The patient apparently recovered from his rheumatic attack, suffered no disturbance from his cardiac disease, except palpitation upon unusual exertion, until finally the whole train of cardiac disease, dyspnæa, pain, and constant palpitation were developed from an hypertrophy of the organ, leading rapidly to a fatal result. He insisted, from these facts, that the physician should never rest satisfied that the heart is uncomplicated in cases of inflammatory rheumatism *because* the patient makes no complaint of *pain in the præcordial region :* the heart should be examined daily. He dwelt upon the pain in this case, (angina pectoris,) as a continued symptom, and accounted for it upon the hypothesis that the inferior cardiac branch of the right pneumogastric had become entangled in the tumor at an early period, subsequently the same thing upon the left side, and reminded the class that the pain from irritation of a nerve is referred to its periphery, and oftentimes to distant parts through its communicating branches.

In describing the value of the physical signs of this case, the differential diagnosis between obstruction of the aortic orifice and imperfect closure of it, was explained as in the one accompanying or masking the *first* sound of the heart heard over its base, and transmitted upwards along the aorta, and in the other heard in the same place, but masking the *second* sound of the heart; is a sound of regurgitation ; is softer and shorter, and seldom, as in this case, heard as far as the apex.

In reference to the hæmoptysis, he called attention to the length of time which had elapsed since its occurrence, and to the case of the celebrated surgeon, Mr. Liston, who died of aneurism five months after the first gush of blood from the trachea, and to another case reported by Dr. Gardner, of Edinburgh, in which hæmoptysis occurred four years and eight months before death; concluding by saying that in this case, it giving no positive evidence of phthisis upon a critical examination, and presenting no proof of disease of the larynx or laryngeal phthisis, and with the positive signs here presented, even in the absence of a tumor, aneurism could be diagnosticated with certainty.

